# Information Filtering on Coupled Social Networks

**DOI:** 10.1371/journal.pone.0101675

**Published:** 2014-07-08

**Authors:** Da-Cheng Nie, Zi-Ke Zhang, Jun-Lin Zhou, Yan Fu, Kui Zhang

**Affiliations:** 1 Web Sciences Center, School of Computer Science & Engineering, University of Electronic Science and Technology of China, Chengdu, People's Republic of China; 2 College of Communication Engineering, Chongqing University, Chongqing, People's Republic of China; 3 Alibaba Research Center for Complexity Sciences, Hangzhou Normal University, Hangzhou, People's Republic of China; 4 Alibaba Research Institute, Hangzhou, People's Republic of China; Semmelweis University, Hungary

## Abstract

In this paper, based on the coupled social networks (CSN), we propose a hybrid algorithm to nonlinearly integrate both social and behavior information of online users. Filtering algorithm, based on the coupled social networks, considers the effects of both social similarity and personalized preference. Experimental results based on two real datasets, *Epinions* and *Friendfeed*, show that the hybrid pattern can not only provide more accurate recommendations, but also enlarge the recommendation coverage while adopting global metric. Further empirical analyses demonstrate that the mutual reinforcement and rich-club phenomenon can also be found in coupled social networks where the identical individuals occupy the core position of the online system. This work may shed some light on the in-depth understanding of the structure and function of coupled social networks.

## Introduction

In the past two decades, the rapid development of Internet has offered unlimited sources for us to search and find out what we need [1]. For instance, we now can enjoy plenty of TV channels as well as countless programs, while only few choices are available twenty years ago. Moreover, the *Internet* not only offers various games, but also becomes a versatile tool to change the lifestyle that we have kept constantly over centuries. For example, online shopping has become more and more popular due to the exponential growth of e-commerce services (e.g. *Amazon.com*, *Ebay.com*, *Taobao.com*, etc), which allow us to choose, compare and purchase goods with single clicks. In addition, there is a vast class of novel job opportunities arising with the emergence of web related applications, such as *SOHO* workers (working at home but communicating via Internet). However, everything has two sides. Although Internet has changed the world a lot and greatly improved our daily life through effectively and efficiently contacting with others, it also brings many side effects and some of which are becoming critically important and even disruptive to our day-to-day routines. One of the most significant dilemmas is the well-known problem of *Information Overload*. Let's take the aforementioned TV programs as an example. In despite of the fact that we indeed enjoy more choices than ever before, it is simultaneously surprising to see that it is even more difficult to find a proper program that is satisfies to us. That is to say, we are facing too many choices to be able to compare them and make the appropriate decisions.

Recently, researchers from various disciplines, including computer science, social science, physics, etc., have devoted much effort to helping users avoid being drowned into the *Information Ocean* [2]. Among numerous applications, the most successful one is the *Search Engine* (SE) [3], whose emergence can be regarded as a milestone. It can help users locate targets by filtering irrelevant objects with designed keywords, hence has soon been widely applied on the Internet. Despite its great success in information filtering, the SE technology also has some apparent drawbacks which interferes its further application in modern human society. On one hand, SE does not consider the personalization of each user, and return exactly the same results for every query with same keywords, regardless of whatever they have searched before [4]. On the other hand, we need to know priori profiles of targets which, however, normally are not very clear for us when the searching is being performed. In addition, sometimes, it is difficult for users to explicitly describe and express their potential intentions in simple words or sentences. So it further increases the difficulty in predicting their underlying preferences. Moreover, SE can only when users proactive submit their queries [5], thus, it lacks the power of actively providing results based on users' searching histories and personalized preferences.

As a consequence, *Recommender Systems* (RS), focusing on mining users' potential options, is considered as a promising candidate to address the excessive sources problem in the information era [6], [7], [8], [9], [10]. RS has achieved a great success in the past few years because it can significantly help users find relevant and interesting items. A recommender system is able to automatically provide personalized recommendations based on the historical records of users' activities. These activities are usually represented by the connections in a user-object bipartite graph [11], [12]. The majority of relevant works in this area can be generally classified into six representative fields: i) Collaborative Filtering (CF) [13], [14]; ii) Content Based Algorithms (CB) [15], [16]; iii) Probability Based Models [17], [18]; iv) Dimension Reduced Approaches [19]; v) Network Based Inference (NB); [12], [20]; vi) Hybrid Algorithms [21], [22]. CF tends to recommend to users with objects that people with similar tastes and preferences favored in the past. There are two categories respectively considering user-based [23] and object-based [14], [24] factors, which should be alternatively applied in different online systems according to their own properties. For instance, *Amazon.com* is a well-known book service provider in which the number of books is more stable than the rapid growth of readers, and thus object-based algorithms could achieve more reliable recommendation results [24]. Comparatively, *Del.icio.us*(http://www.delicious.com/) is a typical user-driven social bookmarking platform [25], hence user-based algorithm is more suitable and effective [26]. Content based methods mainly use text mining techniques to automatically extract out meaningful content and then provide recommendations. Both probability and dimension reduced approaches require much more computational time to obtain the latent variables or vectors [27]. By contrast, network based models, making use of physical dynamics (e.g. random walk [28], [29], [30], heat conduction [20], [31], [32]), try to apply node diffusion process [33] to measure the likelihood of given pair of users and objects to be connected. Such methods would be adjusted to consider the effects of those small-degree (saying *cold*) objects [34], [35] and are especially efficient for recommendation on sparse data sets [36]. Hybrid algorithms do not intend to design new methods but to introduce one or more tunable parameters to integrate different models [37], [22].

Recently, *Social Networks* (SN) [38] have become a powerful tool to characterize various online social services emerging with various Web 2.0 applications [39] in evolutionary games [40], [41], community detection [42] and medical science [43], etc. A great many websites have attracted millions of users active online daily. For example, *Twitter* has more than 1.7 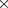 10^8^ users all over the world. *Facebook* has reported to have more than 900 million users registered within two years. *Sina Weibo*, the largest microblogging service provider in China, has been involved by almost 10% of the national population. Therefore, SN provides rich and meaningful social relations to weigh social similarities among users. Therefore, it is expected to be a very useful ingredient to generate more accurate, instructive and explainable recommendation results [44].

**Figure 1:**
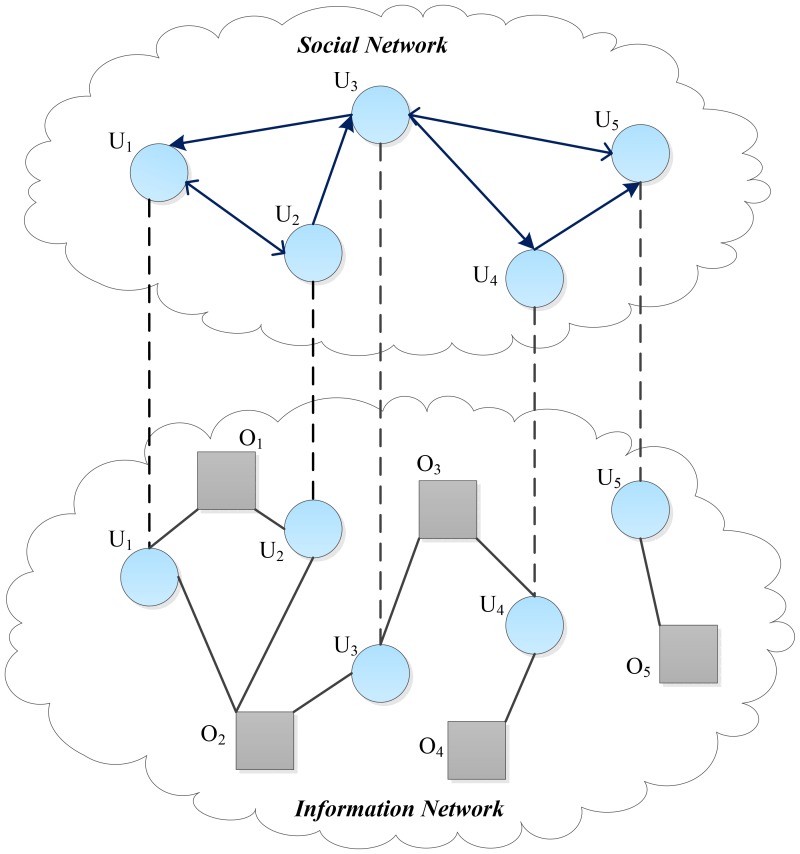
Illustration of a coupled social network with five users and five items, where circles denote users and squares represent objects. (upper layer) social network consists of five users; (lower layer) the information network consists of five objects and five users, while user nodes are the same in the social network.

Coupled networks (CN), also known as interdependent networks [45], contain a joint two-layer network, such as electricity and Internet networks [46], airport and railway networks [47]. There is a kind of coupled nodes, such as cities in the two aforementioned networks, which play the roles of interconnection and maintenance between these two-layer networks [45], [48]. Consequently, those nodes are critically important for the robustness of whole networks [49]. Coupled social networks (CSN), similar with the interdependent networks, also contain such coupling nodes (saying users), which both make friends in the layer of social networks and collect favorites in the layer of information networks. Therefore, those users are especially vital to maintain the structure, connectivity and robustness of social and information networks. Fig. 1 shows an illustration of a simple CSN with five users and five objects. It can be seen that the value of similarity between user 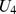 and user 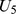 is zero since they do not collect the same object in the information network. So in the traditional complex network theory [50], the relationship between 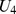 and 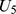 might be considered as irrelevant. However, in fact 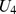 and 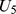 are friends and may have frequent contacts in the social network and they might have many common interests, such as making acquaintance with congenial friends and performing other mutual social activities. Therefore, a comprehensive consideration for the similarity for those two nodes should help improve the consequent recommendation performance. Based on users' distance from a fixed propagation horizon, Massa and Avesani [51] proposed a social propagation method which increased the recommendation coverage while preserving the quality of closeness. Some prior studies also brought social trust and distrust relations to the research of recommender systems [52], [53]. For instance, Knapskog [54], the propagation approach was used to combine pairs of trust and distrust. Bhuiyan [55], the author discussed the definition of trust, and their results demonstrated the positive relationship between trust and interest similarity in online social networks. Crandall [56] proposed a feedback effect between similarity and social influence in online communities. Based collaborative filtering, Esslimani *et al.* [57] proposed a new information network and exploited navigational patterns and transitive links to model users, analyzed behavior similarities, and eventually explored missing links. As we can see, many relationships can constitute a social network such as trust, friendship, community, organizational structure, etc. And some relations are directed, like trust and follower-followee, while others are undirected such as friendship. By utilizing those social relations, we can obtain the strength of social relationship between users, and we can use this weighted social relationship to generate more accurate, explainable and acceptable recommendations though user behavioral information or profiles are unavailable.

The authors [58], [59], [60] have already demonstrated that recommendation performance can be improved by taking into consideration the effect of users' social network. However, how much the effect of social network will take when the social similarity and preference collaboratively work together on recommendation is still unclear. Massa et al. [58], the authors claimed that their purpose is to evaluate the possible contributions of trust-awareness to recommender systems and not to propose a combination technique that would require a dedicated evaluation. Walter et al. [59], the authors presented a model of a trust-based recommendation system on a social network. In their model, agents use their social networks to obtain information and their trust relationships to filter those useless information. However, how to combine the social similarity and preference is still unknown. Zeng et al. [60], the authors designed a social diffusion recommendation algorithm that improves the performance of recommendations. Moreover, they proposed a linear combination of their method and the hybrid method [22]. In this paper, we quantitatively investigate the relationship between social similarity and personal preference for each pair of users through empirical analysis and use a nonlinear method to adjust the effects of them. Therefore, we proposed an algorithm based on CSN by considering the similarities both from social and information networks, and provided recommendations in the classical CF framework. Numerical experiments on two benchmark data sets, *Epinions* and *Friendfeed*, demonstrate that our method can offer more accurate recommendations than previous methods. In addition, extensive analyses show that the RWR-based social similarity can not only enhance the connections between small-degree and large-degree user pairs, but also reveal the large-distance user pairs which cannot reveled by other direct metrics. As a consequence, a wider range of similar users, which cannot be discovered solely from information network, can be made use of to generate more reliable and more precise recommendations.

## Methods

In this section, we start by introducing the approaches to respectively evaluating the social similarity and personalized preference between two users. Then, we integrate them to measure the final similarity of each pair of users, and apply them in recommender systems. Generally, a recommender system consists of two sets, respectively of users 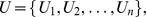 and items 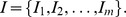 Denote 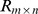 as the adjacent matrix of the user-item bipartite network, of which each element 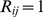 if user 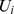 has collected item 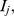 and 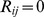 otherwise. Analogously, 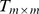 is an asymmetric matrix, denoting the directed social network, where 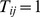 if the user 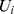 has linked to user 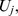 and 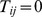 otherwise.

### 1.1 Social Similarity

Firstly, we use the Random Walk with Restart (RWR) [61], [62], [63] method to evaluate the social similarity of directed networks. Consider a random walker starting at node 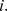 At each step, it can move to 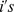 nearest neighbors via directed links with probability 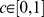 or returns to node 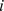 with probability 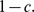 And the final probability of each node at the stationary state will be considered as their respective peer-to-peer influence with node 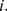 Denote 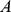 as the transition matrix of the directed network, where 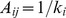 (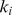 is the out-degree of node 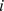 if node 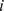 and 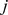 are linked). So, the final probability of *i*'s influence on others can be defined in a vector manner, 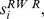 as
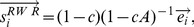
(1)where 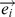 is a unit vector with dimension 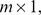 and 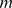 is the number of users. Besides the RWR metrics, we also employ two typical local methods: *LIN* and *LOUT* to evaluate the social similarity, and use the adjusted *Jaccad* method, namely *Tanimoto* coefficient [64], [65], to compute the social similarity between two users. They are defined as:

***LIN***:
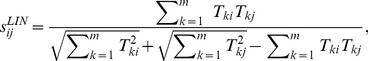
(2)


***LOUT***:
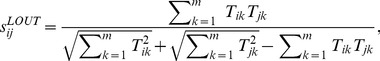
(3)


Then these metrics (Eq. (1)–Eq. (3)) will be used to quantify how much one user influences others. It can be seen that both 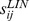 and 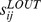 only consider the local information. That is to say, only the common linked nodes of users 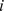 and 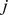 are taken into account. Comparatively, 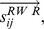 from the perspective of dynamic influence flow, considers both the local and global structure of directed networks. Therefore, it is expected to be a promising index to characterize the social similarity, hence it may provide better a recommendation performance. In addition, when use the Eq. (2)–Eq. (3), we remove the negative value and then normalize the social similarity.

### 1.2 Personalized Preference

There are many methods to compute the common preference between users or items in recommender systems, in which the cosine metric [66] is one of the most frequently used one [67], [68]. It reads as follows:
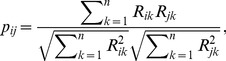
(4)where 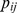 is the examined common preference between nodes 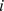 and 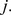


### 1.3 Hybrid Algorithm

To fully make use of the effects made both by influence and preference of users, we adopt a nonlinear hybrid method to integrate them. The final similarity between users 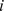 and 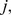 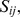 is denoted as
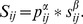
(5)


## Data & Metrics

### 2.1 Data set

In this paper, we use two data sets (datasets are free to download as **Data S1**), *Epinions.com* [69] and *Friendfeed.com* [70], to evaluate the effect of the algorithm. 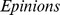 not only allows users to rate items but also permits them to make social connections with others. 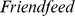 is a microblogging service provider founded in 2007 and acquired by 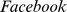 in 2009. To alleviate the sparse problem [71], we purify the two data sets by making sure that each user has at least twenty six out and in-links (2 for 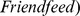 in the social network, and that each user at least collects 7 items (8 items for the 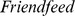 data set) that each item is collected at least 7 times (8 times for 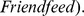 Finally, we obtained a purified data set with 4,066 users, 7,649 items, 217,071 social links and 154,122 bipartite links for 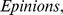 and with 4,188 users, 5,700items, 386,804 social links and 96,942 bipartite links for 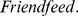 Table 1 shows the basic statistics for two representative data sets).

**Table 1:** Basic properties of the two datasets. 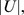 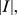 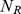 and 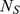 respectively represent the number of users, items, ratings and social activities. 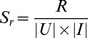 and 
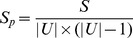
 denotes the data sparsity of information and social networks respectively.

Data sets	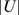	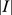	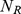	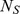	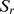	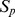
*Epinions*	4,066	7,649	154,122	217,071	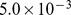	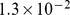
*FriendFeed*	4,188	5,700	96,942	386,804	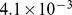	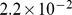

### 2.2 Metrics

Every data set is randomly divided into two parts: the training set which is consisted of 80% of the entries and the testing set consisted of the remaining 20%. For a general recommendation process, the training set is treated as known information to run algorithms and generate corresponding recommendations, yet the information in testing set is unavailable while making recommendations. In addition, we use five metrics to do evaluation in order to fully explore the methods' performance, and we consequently employ five different metrics that characterize recommendation performance:

1. *Precision* [8].– *Precision* represents the probability to what extent a selected item is relevant in a given recommendation list, defined as:
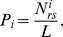
(6)where 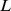 represents the length of recommendation list, and 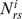 is the number of truly recovered items for user 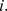 We can obtain the precision of the whole recommender system by averaging over all individuals' precisions,
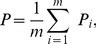
(7)where 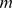 represents the number of users. Obviously, a higher precision means that the algorithm is more accurate.

2. *Recall* [8].— *Recall* represents the probability that a relevant item will be picked from testing set, defined as:
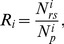
(8)where 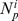 is the number of items collected by user 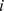 in the testing set, and 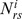 is the number of recovered items of user 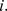 We then obtain the overall recall of the whole recommender system by averaging over all individuals,
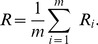
(9)


A higher recall means that the algorithm is more accurate.

3. *F-measure* [8] — The *F-measure* metric is a widely used metric for alleviating the sensitivity of sole usage of precision or recall, defined as,
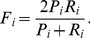
(10)


Anomalously, we can obtain the *F-measure* of the whole system by averaging over all individuals,
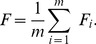
(11)


4. *AUC*— AUC (Area Under ROC Curve) is different from the above three metrics, for *AUC* evaluates the likelihood of all items instead of the *TOP* 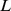 recommendation, where ROC stands for the receiver operating characteristic [72], [8]. It can be approached with a sampling method
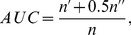
(12)where 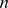 is the number of independent sampling, and 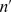 is the number of the predicted score of target item which is higher than that of the randomly selected item, and 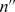 is the times of the target which is the same with random items'. If all the scores are generated from an independent and identical distribution, the then *AUC* should be 0.5. Therefore, how much the value of the *AUC* exceeds 0.5 indicates how much the algorithm performs better than a random prediction.

5. *Diversity (HD)*.— *HD* [22] considers the unique and different user's recommendation list. Given two users 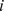 and 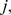 the difference between their recommendations lists can be measured by the Hamming distance.
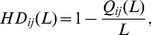
(13)where 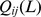 is the number of recommended items in the top-*L* places of both lists. Averaging over all pairs of users' 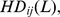 we can obtain the diversity of the observed algorithm. Clearly, higher result (HD) means higher personalization of users' recommendation lists.

## Results & Analysis

### 3.1 Experimental Results

**Figure 2:**
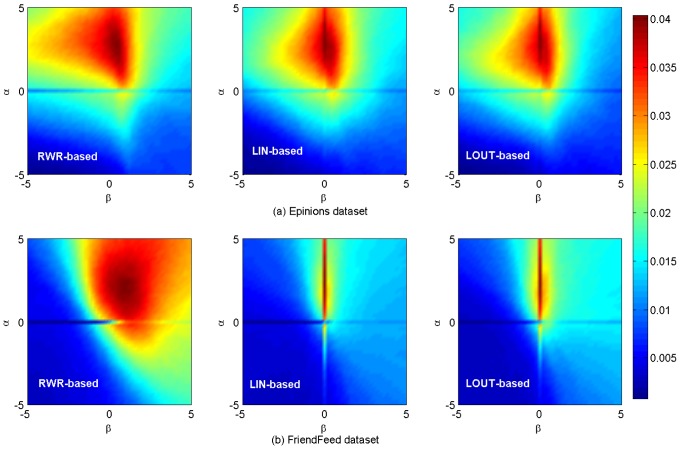
*Precision* results on 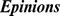 and 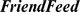 data sets. The length of recommendation list 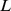 is set as 10.

Fig. 2–Fig. 4 show the algorithm results on 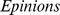 and 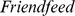 data sets. It can be seen that, for a given length of recommendation list 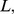 the precision, recall, F-measure and AUC achieve the optimal accuracy for the same parameters for both the LIN-based and LOUT-based method (see also Table 2), which indicates that the local information of both in-flow and out-flow has the similar impact on information filtering. Comparatively, for a moderately small length of recommendation list 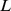 = 10, the precision, recall and F-measure values of RWR-based method reach their maximum value 0.0526, 0.0717 and 0.0512 for 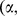 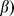 = (2.8, 0.4), respectively. Moreover, the corresponding results are 0.0503, 0.0683 and 0.0489 for 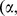 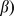 = (3, 0) on 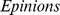 data set whether LIN-based or LOUT-based. For 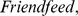 those metrics under RWR-based method have reached 0.0425, 0.1006 and 0.0469 for parameter set 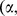 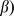 = (2, 0.8), (1.4, 0.8) and (2, 0.8), respectively. For LIN-based or LOUT-based methods, when 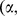 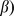 = (2.4, 0), such metrics obtain their maximum value 0.0403, 0.0963 and 0.0443. Similar results can also be found for 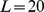 and 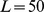 (see Table 2).

**Figure 3:**
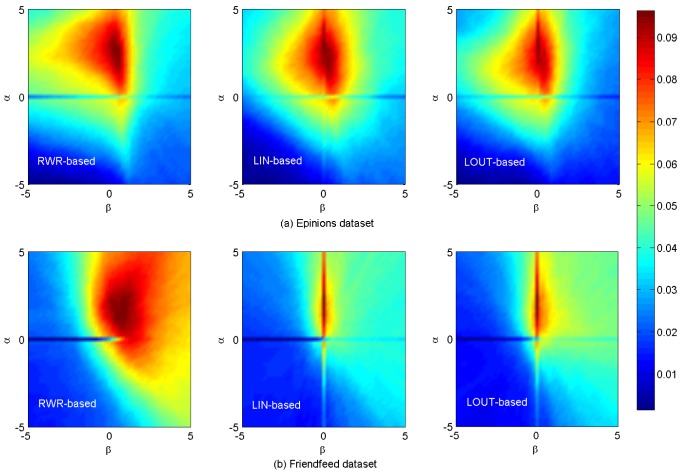
*Recall* results on 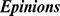 and 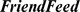 data sets. The length of recommendation list 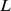 is set as 10.

**Figure 4:**
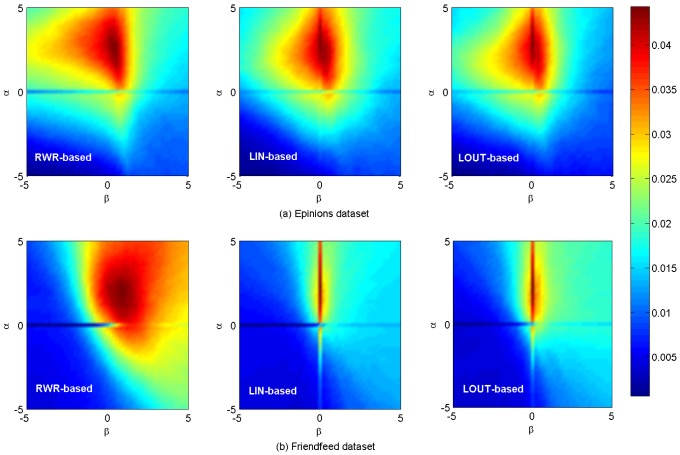
*F-measure* results on 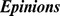 and 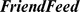 data sets. The length of recommendation list 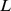 is set as 10.

**Table 2:** Performance of the recommendation algorithms on four metrics: precision (P), recall (R), f-measure (F), and AUC in 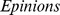 and 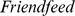 data sets, respectively. 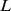 is the length of recommendation list.

		Epinions			FriendFeed		
Method	Metrics	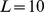	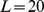	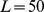	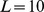	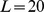	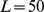
RWR	P	**0.0526**	**0.0402**	**0.0273**	**0.0425**	**0.0325**	**0.0231**
		(2.8, 0.4)	(2.6, 0.4)	(2.8, 0.2)	(2, 0.8)	(1.8, 1.2)	(1.6, 1)
	R	**0.0717**	**0.1076**	**0.1776**	**0.1006**	**0.1507**	**0.2550**
		(2.8, 0.4)	(2.2, 0.4)	(2.4, 0.2)	(1.4, 0.8)	(1.4, 0.4)	(1.6, 1)
	F	**0.0512**	**0.0503**	**0.0426**	**0.0469**	**0.0435**	**0.0370**
		(2.8, 0.4)	(2.6, 0.4)	(2.4, 0.2)	(2, 0.8)	(1.6, 1)	(1.6, 1)
	AUC	**0.7755** (2.4, 0.2)			**0.9053** (0, 2.2)		
LIN	P	0.0503	0.0393	0.0270	0.0403	0.0311	0.0221
		(3, 0)	(3.2, 0)	(2.8, 0)	(2.4, 0)	(2.4, 0)	(2, 0)
	R	0.0683	0.1043	0.1736	0.0963	0.1441	0.2399
		(3, 0)	(2.6, 0)	(2.8, 0)	(2.2, 0)	(1.8, 0)	(2, 0)
	F	0.0489	0.0487	0.0421	0.0443	0.0414	0.0352
		(3, 0)	(3.2, 0)	(2.8, 0)	(2.4, 0)	(2, 0)	(2, 0)
	AUC	0.7729 (2.2, 0)			0.8204 (2.4, 0)		
LOUT	P	0.0503	0.0393	0.0270	0.0403	0.0311	0.0221
		(3, 0)	(3.2, 0)	(2.8, 0)	(2.4, 0)	(2.4, 0)	(2, 0)
	R	0.0683	0.1043	0.1736	0.0963	0.1441	0.2399
		(3, 0)	(2.6, 0)	(2.8, 0)	(2.2, 0)	(1.8, 0)	(2, 0)
	F	0.0489	0.0487	0.0421	0.0443	0.0414	0.0352
		(3, 0)	(3.2, 0)	(2.8, 0)	(2.4, 0)	(2, 0)	(2, 0)
	AUC	0.7729 (2.2, 0)			0.8208 (1.4, 0)		

**Figure 5:**
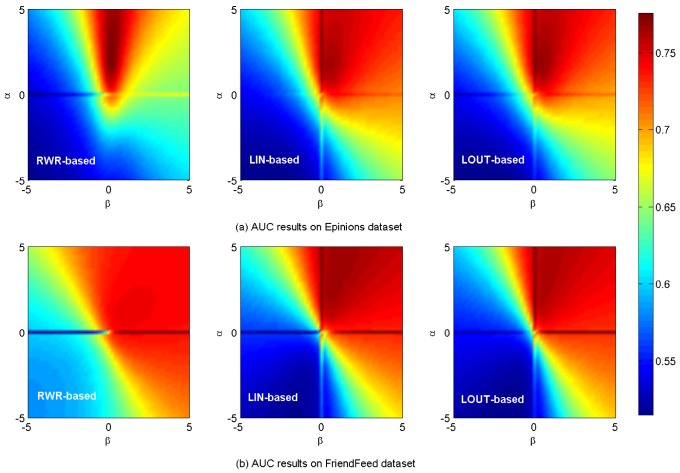
*AUC* results on 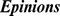 and 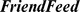 data sets.

**Figure 6:**
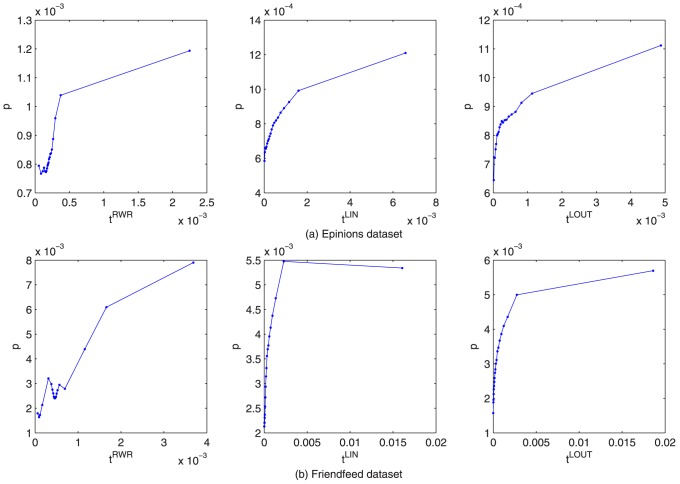
*HD* results on 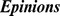 and 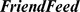 data sets. The length of recommendation list 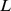 is set as 10.

Fig. 5 shows the *AUC* results. In Fig. 5(a), the maximum *AUC* values are respectively 0.7755, 0.7729 and 0.7729 for 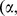 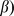 = (2.4, 0.2), 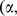 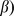 = (2.2, 0) and 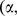 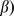 = (2.2, 0) on 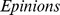 data set. In Fig. 5(b), the corresponding maximum values are respectively 0.9053, 0.8204 and 0.8208 for 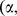 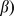 = (0, 2.2), 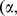 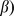 = (2.4, 0) and 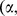 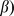 = (1.4, 0) on 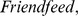 respectively. A brief summary is given in Table 2. Fig. 6 shows the *HD* results on 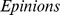 and 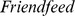 data sets, respectively, and the length of the recommendation list is 10. For all the diversity, their maximum diversity lies in the same position 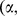 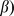 = (5, 5). In Fig. 6 (a), the maximum *HD* values are respectively 0.9864, 0.9817 and 0.9815 for RWR-based, LIN-based and LOUT-based in 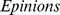 data set. In Fig. 6 (b), the maximum *HD* with RWR-based, LIN-based and LOUT-based, is 0.9928, 0.9923 and 0.9918 for 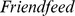 data set, respectively. However, we can find that the diversity in the best *AUC* value's position is higher than that of only using the personal preference. For example, when the recommendation list 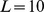 on 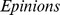 data set, the *HD* values are 0.6944, 0.5297 and 0.4923 in the best *AUC* value's position, only using the personal preference and using the social similarity, respectively.

It is noticed that, for all aforementioned results two crossing lines can be obviously found for LIN- and LOUT-based methods at 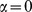 or 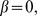 while only a horizontal line is observed for RWR-based method at 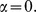 As we known, the cosine, LIN and LOUT are methods for computing similarity simply based on local information, while RWR-based method considers not only the local information, but also takes into account the global social structure. In addition, the behavior network and social network are sparse. Therefore, the personal preference matrix and the social similarity computed by LIN and LOUT might be sparse but the matrix by RWR is full, i.e., there are many zero elements in those matrices that are computed by the cosine, namely LIN, LOUT and RWR. When 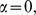 only the social similarity works. Since the personal preference is small, the final similarity will be much sparser. When 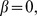 only personal preference works, and the final similarity matrix will be much sparser when using LIN and LOUT methods, i.e., the LIN and LOUT methods will filter the recommendation but the RWR method will supplement it. Thus, that is why it has horizontal lines in the figures and only LIN and LOUT methods have vertical line. As shown in Table 1, the information network is much sparser than that of the corresponding social network, hence more items are possible to be discovered via social connections. In addition, the size of hot areas (corresponding to high performance) of RWR-based method is much larger than that of the other two methods, as it considers not only the nearest neighbors, but also integrates the effect of remote nodes which are not directly connected. Comparatively, the local based (LIN- and LOUT-based) methods can only take into account the commonly direct neighbors, neglecting the global role of each individual. Furthermore, the hybrid case will achieve the best performance for both the observed data sets with optimal parameters 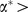 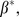 which also proves that social reinforcement is more significant than individual behaviors in information filtering.

**Figure 7:**
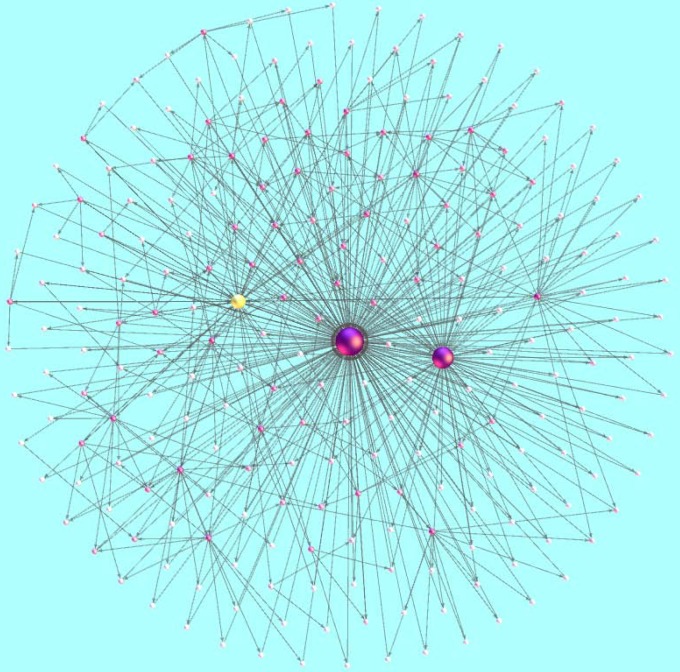
*AUC* results for HHP, BHC and PD methods on 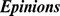 and 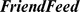 data sets.

Fig. 7 shows that the *AUC* result with one baseline method [22] (HHP for short) and its two variants, [31] (BHC for short) and [30] (PD for short) on 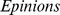 and 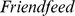 data sets, respectively. It can be seen that the AUC value of HHP method changes monotonously with 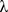 [17], i.e., the HHP method degenerates to pure Mass Diffuse (MD for short) method when 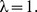 We find that the AUC of both HHP and PD methods increase with 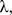 while that of BHC decreases with 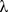 (When 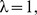 HHP degenerates to the pure MD method, and BHC degenerates to the pure Heat Conduction (HC) method. When 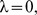 PD degenerates to pure MD method). Generally, the MD method has higher accuracy but lower diversity, while the HC method has higher diversity but lower accuracy. For a better recommendation algorithm, it should ensure higher accuracy principally, thus users might continue to use the system and enlarge their vision by its diverse functions. Therefore, we additionally compare our method with MD. In order to avoid the over-fitting problem [73], we use the three-fold data division [74] to validate our method (see Table 3 and Table 4), where we use 80% of the data as training set, and obtain the optimal parameter value with 10% of the data. We then use the remaining 10% to validate it. It can be seen that the proposed method outperforms the MD algorithm on all the five different metrics.

**Table 3:** Performance of the MD with RWR-based methods obtained under the three-fold data division on 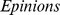 data set. The recommendation list is set as 10.

Methods	precision	recall	f-measure	HD	AUC
MD	0.0275	0.0708	0.0344	0.5999	0.7757
RWR-based	**0.0277**	**0.0723**	**0.0344**	**0.6545**	**0.7975**

**Table 4:** Performance of the MD with RWR-based methods obtained under the three-fold data division on 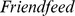 data set. The recommendation list is set as 10.

Methods	precision	recall	f-measure	HD	AUC
MD	0.0254	0.0908	0.0331	0.9258	0.7902
RWR-based	**0.0301**	**0.112**	**0.0397**	**0.9397**	**0.8437**

### 3.2 Empirical Analysis

**Figure 8:**
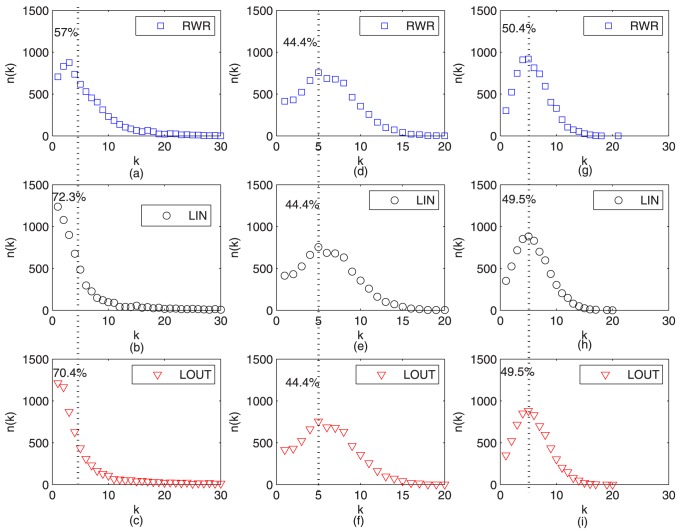
Mean personal preference versus social similarity for 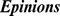 and 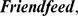 respectively. From left to right, the metrics are respectively RWR-, LIN-, LOUT-based social similarity. The personal preference is averaged according to each social similarity value.

To better understand how the different layers of coupled networks interact with each other, in this section, we empirically investigate the relationship between social similarity and personal preference from micro and macro perspectives. Fig. 8 described that the relationship between social similarity and personal preference for each pair of users. The result shows that, generally, social similarity are positively correlated [55] with personal preference at both local and global measures, indicating that the mutual reinforcement principle [66] also applies to online social activities.

**Figure 9:**
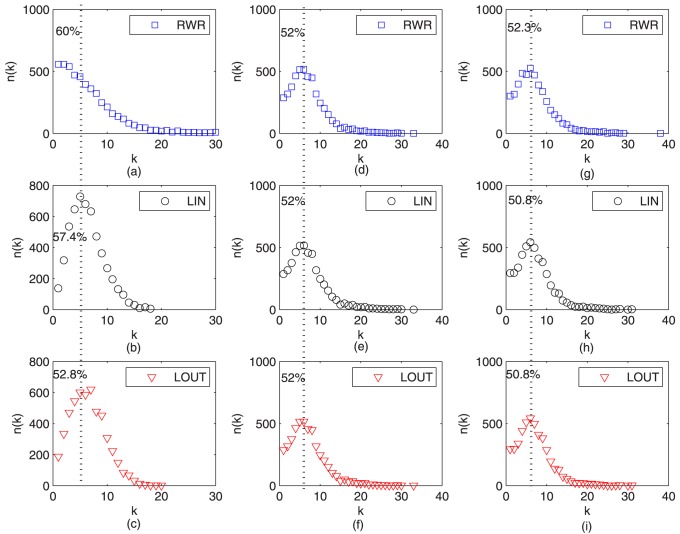
Illustration of a typical example of an ego network for a node with the largest social similarity value (the biggest size).

**Figure 10:**
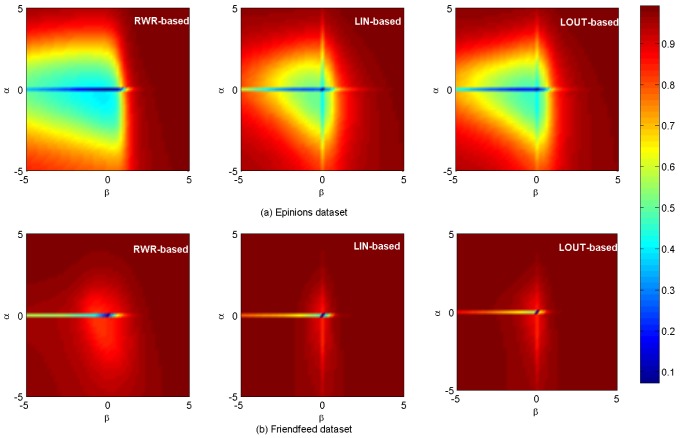
Number of recommended items versus degree on *Epinions* for 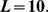 From left to right, the parameters 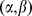 of Eq. (5) are set as (1,0), (0,1), and 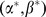 given in Table 2, respectively. The dash line indicates the degree of 5, and the corresponding number shows the percentage of all the recommendation items.

**Figure 11:**
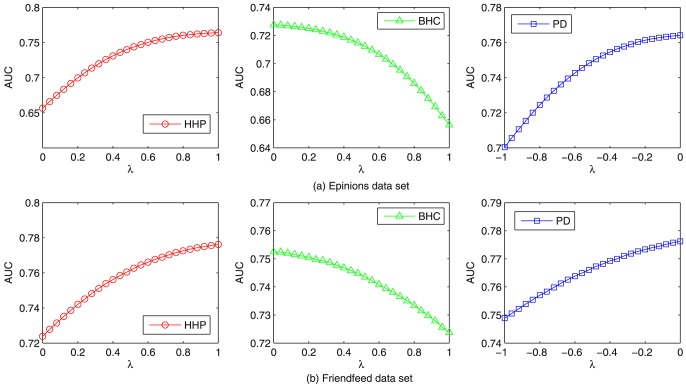
Number of recommended items versus degree on *FriendFeed* for 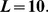 From left to right, the parameters 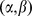 of Eq. (5) are set as (1,0), (0,1), and 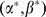 given in Table 2, respectively. The dash line indicates the degree of 5, and the corresponding number shows the percentage of all the recommendation items.

In Fig. 9, we also find that a typical example of an ego network [75] for a node with the largest social similarity value (with the biggest size). It can be seen that it connects to a node of relatively large social similarity yet small similarity (yellow one), suggesting the rich-club phenomenon [76] of social interests activities. That is to say, users with high social impact tend to interact with users of high social similarity, even if they lack common activities. Furthermore, we also find that the degree distribution of successfully recommended items in Fig. 10 and Fig. 11 for Epinions and Friendfeed, respectively. In Fig. 10(a–c) and Fig. 11(a–c), the parameters of Eq. 5 are set as 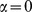 and 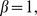 of which only the social similarity takes effect in the recommendation process. It shows that the local measures (LIN and LOUT) are more likely to to find small-degree items (the degree is smaller than 5) than the RWR metric (around 57%). Similarity, for another extreme case of Eq. 5, 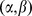 is set as (1,0), implying that only the personal preference will work for information filtering, hence all the results are identical in Fig. 10(d–f) and Fig. 11(d–f), respectively. In addition, the number of recommended small-degree items is fewer than that of social based method. Comparatively, in Fig. 10(g–i) and Fig. 11(g–i), the parameter 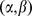 is set as the optimal case given in Table 2. Since both the social similarity and personal preference are integrated, the hybrid algorithm not only can find those *cold* items [34], [26] (where the social similarity primarily works), but also can push some popular items (which is largely because of the personal preference). Therefore, it finally can achieve a better performance for information filtering. In addition, the novelty [10] of recommender systems refers to how different the recommended objects are from what the users have already seen before. The simplest way to quantify the ability of an algorithm to generate novel and unexpected results is to measure the average popularity of the recommended objects. The lower the average objects's degree in the recommendation list, the better the novelty of the system. From Fig. 10 and Fig. 11, we can see that the number of recommended small-degree items is larger than that of only using personal preference and fewer than that of the social based method, i.e., our method has higher novelty than that of only using personal preference.

## Conclusions & Discussion

In this paper, we have proposed a hybrid information filtering algorithm based on the coupled social networks, which considers the effects of both social similarity and personalized preference. We apply three metrics, *LIN*, *LOUT* and *RWR*, to evaluate the asymmetrically social similarity, and use the cosine similarity to measure the symmetrically personalized preference. In addition, we integrate them with two tunable parameters in order to obtain better recommendation results. Experimental results show that the hybrid pattern can not only provide more accurate recommendations, but also enlarge the recommendation coverage while adopting global metric (RWR). Further empirical analyses demonstrate that the mutual reinforcement can also be extended to coupled networks where the same individuals occupy the core position of the entire online society. However, this article only provides a simple start for making use of both behavior and social information, while a couple of issues remain open for future study. Especially, the underlying mechanism driving the interaction of social and information networks is of particular importance to deeply understand how coupled social networks work, as well as its potential applications.

## Supporting Information

Data S1 **Datasets.**
(ZIP)
